# Effects of Two Sublethal Concentrations of Mercury Chloride on the Morphology and Metallothionein Activity in the Liver of Zebrafish (*Danio rerio*)

**DOI:** 10.3390/ijms17030361

**Published:** 2016-03-11

**Authors:** Rachele Macirella, Antonello Guardia, Daniela Pellegrino, Ilaria Bernabò, Valentina Tronci, Lars O. E. Ebbesson, Settimio Sesti, Sandro Tripepi, Elvira Brunelli

**Affiliations:** 1 Department of Biology, Ecology and Earth Science, University of Calabria, Via P. Bucci 4/B, Rende (Cosenza) 87036, Italy; rachele.macirella@unical.it (R.M.); antonello.guardia@unical.it (A.G.); danielapellegrino@unical.it (D.P.); ilaria.bernabo@unical.it (I.B.); settimio.sesti@unical.it (S.S.); sandro.tripepi@unical.it (S.T.); 2 Uni Research Environment, Uni Research, Bergen 5006, Norway; valentina.tronci@uni.no (V.T.); smoltbrain@me.com (L.O.E.E.); 3 Department of Biology, University of Bergen, Bergen High Technology Center, Bergen 5020, Norway

**Keywords:** inorganic mercury, liver, acute effects, histology, ultrastructure, confocal microscopy, *in situ* hybridization, metallothionein

## Abstract

Mercury (Hg) is a highly hazardous pollutant widely used in industrial, pharmaceutical and agricultural fields. Mercury is found in the environment in several forms, elemental, inorganic (iHg) and organic, all of which are toxic. Considering that the liver is the organ primarily involved in the regulation of metabolic pathways, homeostasis and detoxification we investigated the morphological and ultrastructural effects in *Danio rerio* liver after 96 h exposure to two low HgCl_2_ concentrations (7.7 and 38.5 μg/L). We showed that a short-term exposure to very low concentrations of iHg severely affects liver morphology and ultrastructure. The main effects recorded in this work were: cytoplasm vacuolization, decrease in both lipid droplets and glycogen granules, increase in number of mitochondria, increase of rough endoplasmic reticulum and pyknotic nuclei. Pathological alterations observed were dose dependent. Trough immunohistochemistry, *in situ* hybridization and real-time PCR analysis, the induction of metallothionein (MT) under stressor conditions was also evaluated. Some of observed alterations could be considered as a general response of tissue to heavy metals, whereas others (such as increased number of mitochondria and increase of RER) may be considered as an adaptive response to mercury.

## 1. Introduction

Mercury (Hg) is a naturally occurring metal derived from several processes, such as weathering of the earth’s crust, soil erosion, geologic activities, and its contents in the environment can vary between different regions [1]. Human sources of mercury pollution include activities such as agriculture, municipal wastewater discharges, mining, incineration, and discharges of industrial wastewater [2].

These primary sources both natural and anthropogenic, have been directly mobilized by humans for thousands of years into aquatic and terrestrial ecosystems [3] resulting in an increased release into the environment that often exceed permissible levels.

Mercury has drawn the attention of scientific community since the 1950s when the first evidences of dangerous environmental effects have been reported [4], thus prompting policies and regulations to limit Hg emissions. Recently the World Health Organization (WHO) has ranked mercury as one of the 10 chemicals of major public health concern worldwide [5].

Mercury (Hg) is a highly hazardous pollutant exceedingly bioaccumulative and is commonly found in the environment in several forms, elemental, inorganic (iHg) and organic, all of which are toxic [6]; however the principal natural and anthropogenic emissions of mercury exist as inorganic forms [4].

With regard to mercury pollution, particular attention has been paid to aquatic environment, and mercury is considered as priority hazardous contaminant for fishes and wildlife [4]. Moreover, due to their tendency to bioaccumulate and biomagnify through the food chain, mercury compounds represent substances of very high concern for human health. It has been demonstrated that exposure to mercury, both inorganic and organic, may cause serious damage in both human and experimental animals [7,8,9,10]. The critical target organ for mercury is the nervous system but it can impair any organ [11].

In fishes it has been demonstrated that mercury affects the brain [12,13,14], muscles [15,16], immune systems and immunoresponse [17], and the reproductive system [18]. It has also been shown that mercury is able to induce structural degeneration of cells, oxidative stress, variations of energy metabolism and calcium homeostasis [19,20].

The liver is the organ primarily involved in the regulation of metabolic pathways, homeostasis and detoxification [21,22], therefore toxicity induced by mercury can affect its morphology, and impair its functional role by interfering with key physiological and metabolic processes.

Several authors have tested different mercury concentrations administered through diet, ambient water or intraperitoneal injection, using both acute and chronic tests; different parameters have been used to assess the effect of this pollutant on fish liver (*i.e.*, gene expression, proteomic, transcriptome analysis, DNA microarray, biochemical) [23,24,25,26,27,28,29], but very few studies have focused on histological and ultrastructural alterations [9,22,30,31]. Studies on mercury have mainly focused on methyl mercury (MeHg) [9,13,14,15,23,30], and the iHg effects have been substantially underestimated despite the evidence that this form mainly accumulates in the kidney and in the liver of fishes [32,33,34].

In this work we used as model zebrafish (*Danio rerio*), a species that has recently been used in an increasing number of toxicological studies. The small body size, the easy husbandry, the short generation time and the early morphology make this species an ideal model for toxicological research aimed to clarify the effects of chemical exposure [35].

A number of studies have examined the accumulation, uptake and localization of both organic and inorganic mercury in zebrafish embryos and larvae using synchrotron X-ray fluorescence mapping [36,37,38]. Only few papers investigated the effects of iHg on *Danio rerio* liver and there is a general paucity of information about morphological and functional effects of mercury in fish liver. A recent reports have examined the implications of MeHg and iHg exposure on the toxicity mechanisms in *Danio rerio* muscle, liver and brain after 7, 25 and 62 days of dietary exposure [9].

To date only one study [22] has addressed how iHg can affect *Danio rerio* liver morphology after exposure to 50 and 200 μg/L of water dissolved HgCl_2_.

There may be different mechanisms through which the toxicity of mercury can act on liver and a wide range of biological responses can be useful as biomarkers for Hg contamination.

In ecotoxicological studies the use of morphological and functional endpoints approach is recommended for a more reliable assessments of biochemical responses after exposure to different environmental stressors [39,40].

The MTs are proteins able to absorb toxic substances and for this reason they have been elected as excellent biomarker in the assessment of metal exposure and prediction of potential detrimental effects induced by metal contamination [41,42]. The induction of MT biosynthesis in fishes after metal exposure is well established [43,44] and in particular mercury is considered an effective inducer of metallothionein (MT) synthesis [45].

Hence in order to have a more comprehensive overview of iHg-induced effects, we evaluated histology, ultrastructure and MTs expression in *Danio rerio* liver after acute exposure to two low HgCl_2_ concentrations with the aim of further elucidating the role of morphofunctional studies in monitoring aquatic environment.

## 2. Results

### 2.1. Morphology and Ultrastructure

#### 2.1.1. Control

The morphology and ultrastructure of *Danio rerio* liver have been previously described [46,47] and only a brief general description relevant to the present work will be given. *Danio rerio* liver is a dense organ divided into three lobes, located in the cranial part of abdominal cavity surrounding the digestive tract. In this species hepatic lobules, portal areas and hepatic arteries that are characteristics of liver, cannot be observed. The blood enters in the liver trough the portal vein that branches directly into the sinusoids. The sinusoids converged to the central vessel and the blood is carried out of liver.

**Figure 1:**
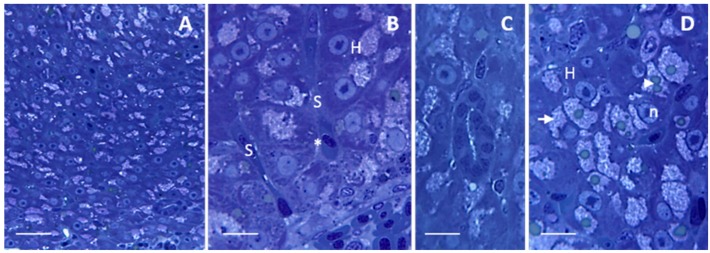
Light micrographs showing *Danio rerio* liver under control conditions. (**A**) Homogeneous organization of liver parenchyma. Bar 25 μm; (**B**) Hepatocytes (H) surrounding the sinusoids (S) are disposed in cords. The space of Disse (asterisk) lies between the hepatocytes and the sinusoidal endothelium. Bar 10 μm; (**C**) Bile duct enclosed by cuboidal epithelium. Bar 10 μm; (**D**) Hepatocytes contain several (arrowhead) lipid droplets surrounded by numerous glycogen granules (arrow). n = nucleus. Bar 10 μm. All toluidine blue.

Under light microscope, liver shows a homogeneous parenchyma composed of hepatocytes (Figure 1A) surrounding a network of sinusoids. Hepatocytes that surround the sinusoids are disposed in two rows that give rise to cords (Figure 1B); the sinusoidal lumen is lined by a continuous endothelium where no basal lamina occurs (Figure 1B). The parenchyma is well-drained by a reticulum of circular structures called bile duct that are enclosed by cuboidal epithelium (Figure 1C). The space of Disse (also called perisinusoidal space) extends between the sinusoidal endothelium and the hepatocytes (Figure 1B). Hepatocytes show uniform size and contain a big nucleus located in the center of the cell; a dense nucleolus is usually evident (Figure 1B,D). Within the cytoplasm lipid droplets, often surrounded by numerous glycogen granules, could be observed (Figure 1D).

**Figure 2:**
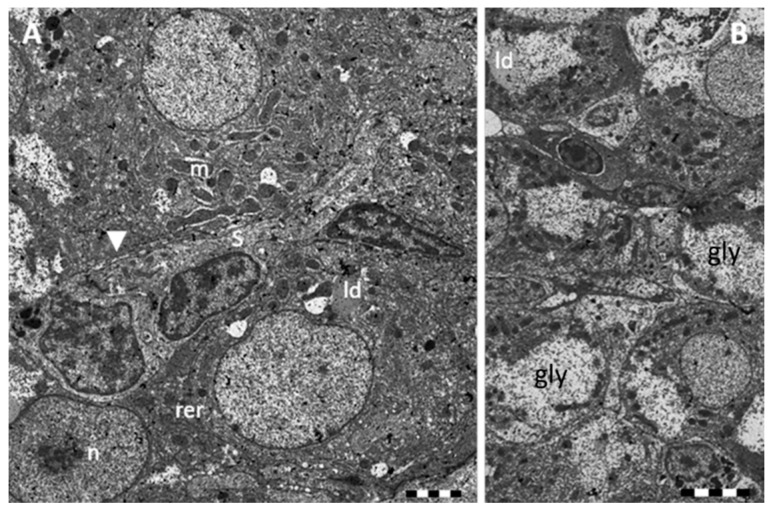
TEM micrographs showing *Danio rerio* liver section under control conditions. (**A**) High resolution of hepatocytes surrounding a sinusoid; arrowhead = space of Disse. m = mitochondria; n = nucleus; rer = rough endoplasmic reticulum. Bar 2 μm; (**B**) Lipid droplets (ld) and glycogen reserves (gly) are abundant in the cytoplasm of hepatocytes. Bar 5 μm.

Observed under TEM, the space of Disse appears as a region of low electron-density (Figure 2A).

The cytoplasm of the hepatocytes shows an high grade of electron-density and it is characterized by a well-developed rough endoplasmic reticulum (RER) that often runs parallel to the cellular membrane and nuclear membrane (Figure 2A). Numerous mitochondria of different shape (spherical and oval forms) are scattered through the cytoplasm (Figure 2A,B); they show few cristae and some electron-dense glycogen granules. Golgi complex is poorly developed and smooth endoplasmic reticulum (SER) is not appreciable. A large amount of low density glycogen granules are mainly gathered at the periphery of the hepatic cytoplasm (Figure 2B); several small lipid droplets of different size can also be observed (Figure 2A,B). Nucleus is spherical or ovoid and is usually located in the center of the hepatocyte; the granular euchromatin occupies the most part of the nucleus whereas the heterochromatin is distributed at the periphery; an highly stained nucleolus is also observed, often in the central region of the nucleus (Figure 2A,B).

#### 2.1.2. Exposed Fishes

**Figure 3:**
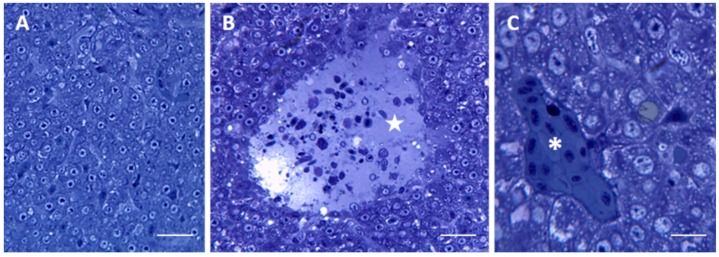
Light micrographs of *Danio rerio* liver section after 96 h of exposure to 7.7 μg/L HgCl_2_. (**A**) General appearance of liver morphology after 96 h of exposure to 7.7 μg/L HgCl_2_. Bar 25 μm; (**B**) In the liver parenchyma lysed area (star) are visible. Bar 25 μm; (**C**) The cytoplasm of hepatocyte appears vacuolated. It is possible to see some occluded vessels (asterisks). Bar 10 μm. All toluidine blue.

After 96 h of exposure to 7.7 μg/L of HgCl_2_ (for simplicity, we will refer to 7.7 μg/L as the “low” concentration), the histological observation reveals severe alteration in liver morphology of treated animals compared with control (Figure 3A). The liver parenchyma undergoes degenerative changes and it is possible to observe large areas of lysis (Figure 3B) whereas sinusoids and venules are filled with red blood cells (Figure 3C).

The major changes observed in the hepatic parenchyma of exposed fishes include an intense cytoplasm vacuolization and a decrease in the content of both lipid droplets and glycogen granules (Figure 3C).

**Figure 4:**
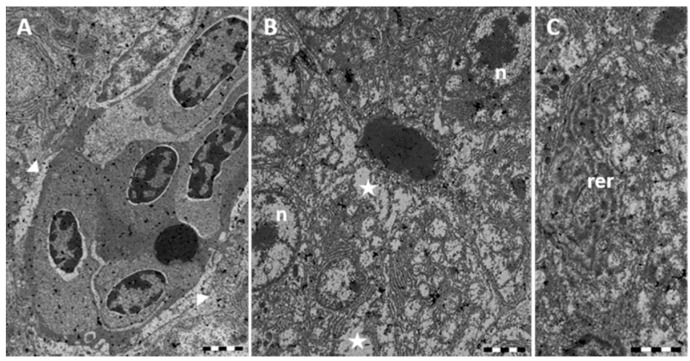
TEM micrographs of *Danio rerio* liver section after 96 h of exposure to 7.7 μg/L HgCl_2_. (**A**) Alterations in the Disse’s space (arrowheads); (**B**) In the hepatocyte large cytoplasmic lacunae (star) are detected; nuclei (n) appear damaged; (**C**) The rough endoplasmic reticulum (rer) is considerably dilated and fragmented. Bar 2 μm.

Ultrastructural observations reveal the modification of the Disse’s space architecture with alterations to the endothelial cell and congestion of blood vessels (Figure 4A). TEM observations confirm the conspicuous vacuolization of the hepatocytes cytoplasm that shows a wide electron-clear area (Figure 4B). It is also evident the dilation and fragmentation of RER cisternae and the decrease of glycogen reserves (Figure 4B,C). Nuclei often lose their rounded shape and show a change in both distribution and amount of heterochromatin (Figure 4B). Cytoplasmic organelles are no longer distinguishable (Figure 4B,C).

**Figure 5:**
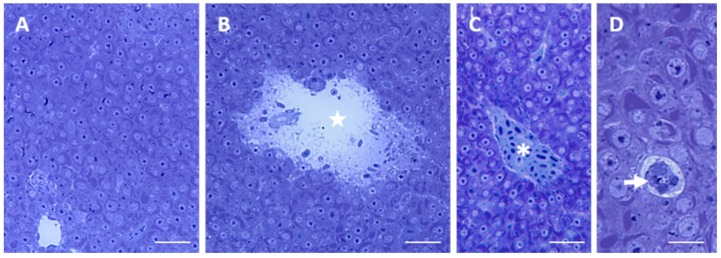
Light micrographs of *Danio rerio* liver section after 96 h of exposure to 38.5 μg/L HgCl_2_. (**A**) General appearance of liver morphology after 96 h of exposure to 38.5 μg/L HgCl_2_. Bar 25 μm; (**B**) Big lysate area (star) in the liver parenchyma. Bar 25 μm; (**C**) Some blood vessels are occluded (asterisk). Bar 25 μm; (**D**) Degenerating cell (arrow). Bar 10 μm. All toluidine blue.

After 96 h of exposure to 38.5 μg/L of HgCl_2_ (for simplicity, we will refer to 38.5 μg/L as the “high” concentration), the liver morphology appears markedly altered. (Figure 5A) In the liver parenchyma, conspicuous areas of lysis are evident (Figure 5B) along with vessels congestion (Figure 5C).

In the hepatic parenchyma of exposed fishes, cytoplasm vacuolization is not detected and the most evident feature is the presence of a large and extended RER at the periphery of the nucleus (Figure 5D). A decrease in the content of both lipid droplets and glycogen granules can also be detected (Figure 5C,D). Within the parenchyma several degenerate hepatocytes are observed (Figure 5D).

**Figure 6:**
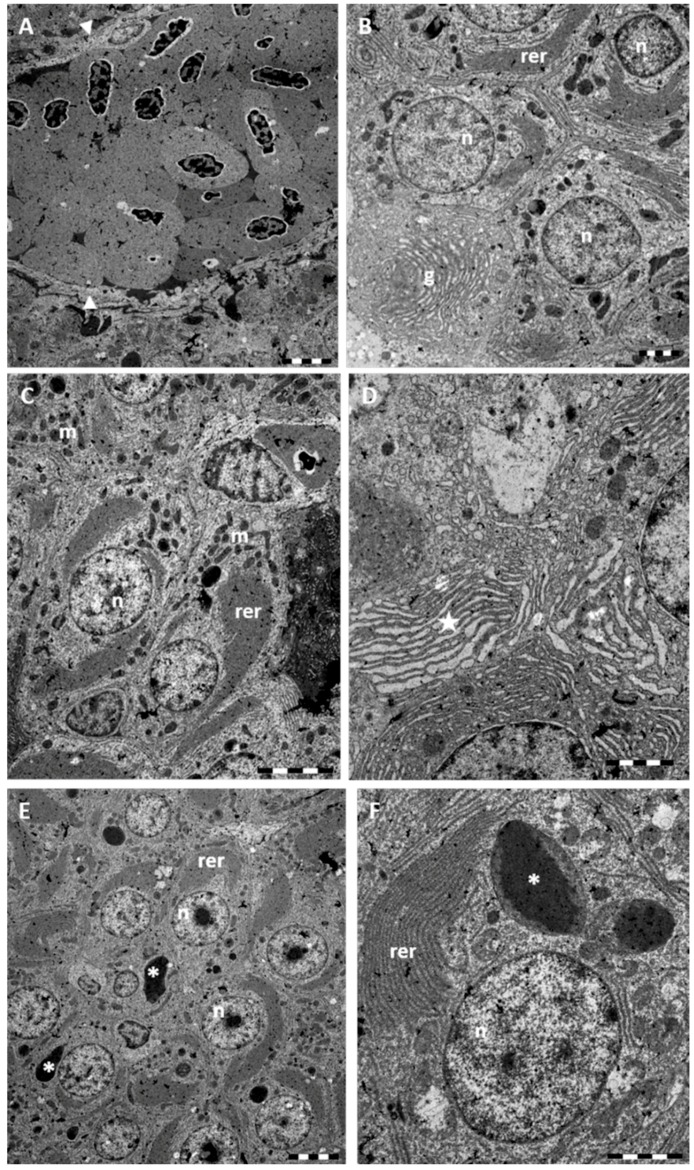
TEM micrographs of *Danio rerio* liver section after 96 h of exposure to 38.5 μg/L HgCl_2_. (**A**) Occlusion of blood vessel and damaged Disse’s space (arrowheads). Bar 5 μm; (**B**) RER appear conspicuous and fills most part of the cytoplasm. Note the well-developed Golgi apparatus (g). Some degenerate hepatocyte with pyknotic nuclei can be seen. Bar 2 μm; (**C**) Numerous mitochondria (m) are scattered through the cytoplasm. Bar 5 μm; (**D**) High magnification of RER; note at several points, swelling of cisternae (star). Bar 2 μm; (**E**) In the central portion of nuclei a large nucleolus is evident. In the cytoplasm we can observe the abundant RER and the presence of large electron-dense granules (asterisk) of variable dimension. Bar 5 μm; (**F**) High magnification of atypical granules (asterisk). Bar 2 μm. n = nucleus; rer = rough endoplasmic reticulum.

Under TEM, it is possible to detect the morphological alterations of Disse's space, and presence of numerous red blood cells filling sinusoids and venules (Figure 6A). Ultrastructural observations provided further evidence of the great development of RER which occupies large parts of the electron-clear cytoplasm of hepatocytes (Figure 6B–F). A conspicuous Golgi apparatus with curved cisternae can also be seen (Figure 6B). The numerous sparse mitochondria show a slightly increased electron-density (Figure 6B,C). In the central portion of the nucleus a conspicuous nucleolus can often be seen whereas heterochromatin concentrates at the periphery (Figure 6B–F). Scattered through the liver parenchyma several degenerating hepatocytes can be detected (Figure 6C). At several point a swollen RER cisternae, and pyknotic nuclei are evident (Figure 6B,C). In the cytoplasm, atypical cytoplasmic electron-dense granules are often detected (Figure 6C,E).

### 2.2. In Situ Hybridization

**Figure 7:**
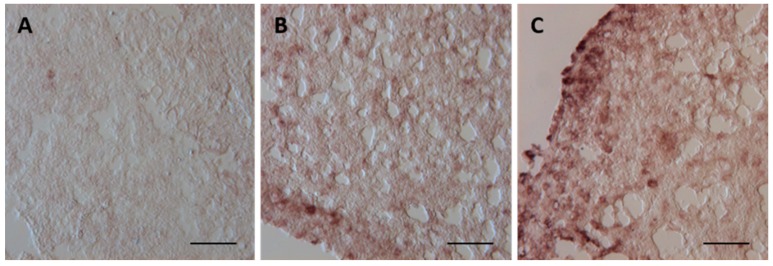
*Mt* mRNA expression assessed by *in situ* hybridization on *Danio rerio* liver cryosections. (**A**) Weak *mt* mRNA hybridization signal in the control samples; (**B**). After 96 h of exposure to 7.7 μg/L of HgCl_2_, the cytoplasmic areas of the hepatocytes show positive hybridization signal; (**C**) Increase in hybridization signal in liver from animals exposed to 38.5 μg/L of HgCl_2_. All bar 45 μm.

In the control group the hybridization signal is too weak to allow confident identification of the cells expressing *mt* mRNA (Figure 7A).

Compared to the control group, a positive hybridization signal is observed in liver of specimens exposed to the low concentration. In particular, a marked increase in signal development in the whole parenchyma is detected with a peak in the cytoplasmic areas of the hepatocytes (Figure 7B). In specimens from the high concentration group, the transcript observed in the liver parenchyma shows a slightly higher intensity compared to that detected in the low concentration group: the signal is observed in all liver tissue and is more intense at the periphery of parenchyma (Figure 7C).

### 2.3. Immunolocalization of MTs

**Figure 8:**
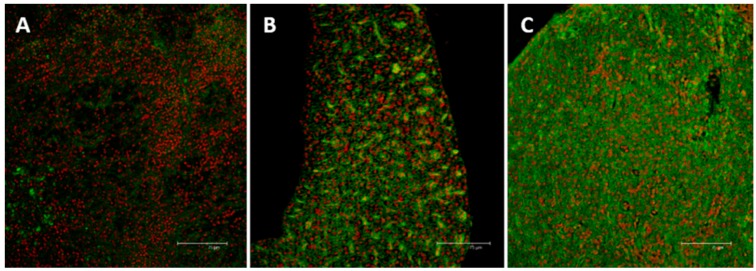
Confocal micrographs of *Danio rerio* liver sections labeled with a mouse monoclonal antibody against MT (**green**—FITC labeled); nuclei labeled with propidium iodide (**red**). (**A**) Control samples revealed no or weak signal in few hepatocytes; (**B**) After 96 h of exposure to 7.7 μg/L of HgCl_2_, MT immunoreactivity could be observed in hepatocytes cytoplasm; (**C**) After 96 h of exposure to 38.5 μg/L of HgCl_2_ the intensity of staining increased and signal was mainly detected in damaged areas. All bar 75 μm.

In the liver of animals from control group very few labeled cells show a weak immunoreactivity for MTs (Figure 8A). After 96 h of exposure to the low concentration, the immunohistochemical analysis reveal a significant increase in MTs labeling; a pronounced MTs immunoreactivity can be observed in the whole liver parenchyma (Figure 8B). After 96 h of exposure to the high concentration, MTs labeling increases; immunoreactive cells are distributed throughout the liver parenchyma (Figure 8C).

### 2.4. Gene Expression

**Figure 9:**
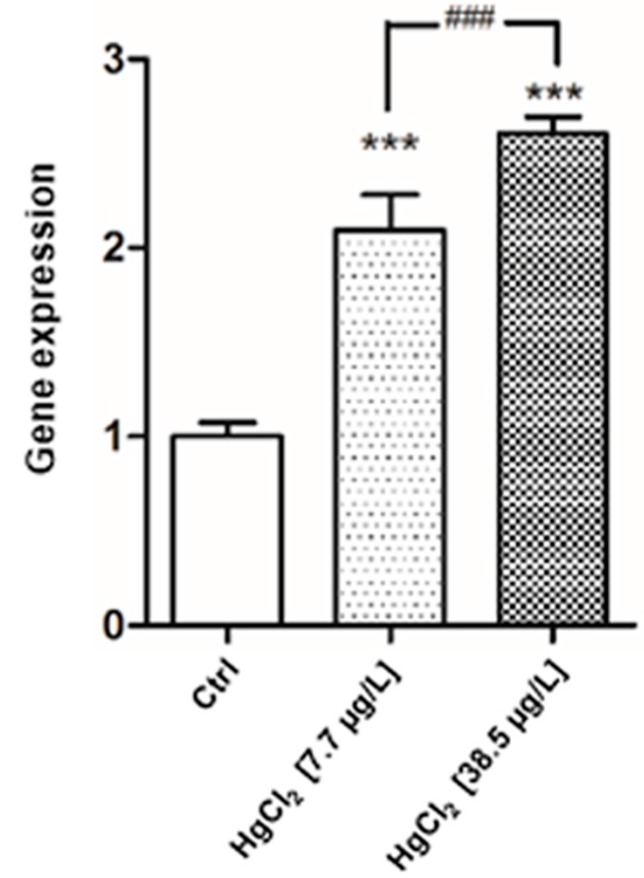
Relative variations in *mt* Gene Expression As Compared to control in *Danio rerio* liver after 96 h of exposure to HgCl_2_ (7.7–38.5 μg/L). Taqman real time relative quantitative PCR. The bars show mean ± S.D, *n* = 5. Asterisks indicate the treated groups that differ from the control, *** *p* < 0.001; hashtags indicate difference between treated groups ^###^ *p* < 0.001 (One way ANOVA followed by Bonferroni’s post hoc test).

The genetic analysis evidenced a tissue-specific expression rate. In addition to the basal genetic expression of *mt* in liver, the gene shows a significant modification of its expression in both treatments after 96 h of exposure to HgCl_2_ (Figure 9). *Mt* gene is significantly increased (*p* < 0.001) at 7.7 μg/L exposure level compared to control group; the gene shows the highest responses at 38.5 μg/L of HgCl_2_ when compared to basal condition (*p* < 0.001).

## 3. Discussion

Our observation on healthy *Danio rerio*, confirm the previously described liver organization [46,47]; the homogeneous parenchyma is composed of hepatocytes, arranged in cords, surrounding a network of sinusoids that converged to the central vessel. Hepatic lobules, portal areas, and hepatic arteries were not seen.

In the present study we investigated the effects of HgCl_2_ on zebrafish liver parenchyma through a morphofunctional and ultrastructural study; we showed that liver injury was induced when fishes were exposed to mercury chloride for 96 h at doses of 7.7 and 38.5 μg/L; the doses of mercury administered in our experiments were very low, but high enough to produce severe liver injury and also inducing an enhanced expression of hepatic MTs. These effects was dose dependent.

Examination of liver after exposure to both tested concentrations showed remarkable effects, resulting in modifications in both histology and ultrastructure.

The dilation of Disse’s space and the occurrence of wide lysed areas were the most evident result of Hg application. Morphological disorders and necrosis in the Disses’s space were found in *Hoplias malabaricus* after chronic exposure to dietary methylmercury [30]. These authors suggested that endothelial cells alterations may affect the exchange between hepatocytes and sinusoids (e.g., nutrients, chemical signals and physiological components) thus resulting in the disturbance of cell physiology and organ function stability.

The appearances of lysed areas were found in zebrafish liver after exposure to copper sulfate [48]. Considering the copper’s ability to induce membrane disruption, these authors suggested that the lysis distribution may reflect a heterogeneous distribution of Cu in the parenchyma. Interestingly, we observed the appearance of lysed area after exposure to iHg; whereas similar adverse effects have not been reported in liver after exposure to MeHg; these results further support the hypothesis that iHg, affect membrane whereas methylmercury does not appear to act directly at plasma membrane [22,49].

In the present study, a drastic decrease in glycogen amount and lipid granules was observed in the hepatocyte of both low and high exposed groups.

Glycogen depletion has been reported in liver fishes, after short and long term exposure to other heavy metals, such as copper, cadmium and lead [48,50,51]. Moreover, similar glycogen perturbations have also been recorded in both liver and muscle of *Oreochromis mossambicus* as result of mercury chloride toxicity; Roy George and colleagues [27] suggested that the great loss of glycogen in the liver indicates that it is the most affected organ during stress. Since the liver is the main site for detoxification, glycogen depletion could be correlated with the increased glycolytic activity required by enhanced metabolism under stress conditions.

In the hepatocytes the presence of glycogen granules and lipid droplet is related to the normal metabolic function of the cells. In addition, the depletion of lipids described in our work is often observed in fishes after exposure to both organic and inorganic mercury [31,52]. The depletion of both reserve substances may suggest an increase of basal metabolic pathways of hepatocytes, due to the histological modification after experimental conditions.

After exposure to low HgCl_2_ concentration, other common ultrastructural alterations observed in zebrafish hepatocytes were: organelle disorganization within cytoplasm (empty appearance), cytoplasmic vacuolization, RER dilation and fragmentation. Similar morphological alterations were also found after exposure to other heavy metal suggesting an unspecific adaptive response of the liver to stress [48]. Interestingly, some ultrastructural alterations observed after the exposure to high Hg concentration such as increased number of mitochondria and increase of RER, seems to be adaptive. The great development of RER indicate an enhanced hepatic protein synthesis [48].

The electron dense granule in the cytoplasm along with the increase of heterochromatin in hepatocytes nuclei have been reported after exposure to mercury in *Hoplias malabaricus*. The authors suggested that this morphological alteration of nuclei is evidence that this organelle can accumulate metals more intensely than other cellular compartments [30].

Numerous studies have demonstrated how the metallothioneins are important in the regulation of biological functions such as homeostasis of zinc and copper [53,54], metal detoxification [55,56] and oxygen radical scavenging [57,58]. The MTs have been elected in both vertebrates and invertebrates as a biomarker of environmental metal pollution [59,60,61,62]. Based on the strong correlation between MTs expression and environmental concentrations of metals, the amount of MTs in the cells is considered essential for monitoring the exposure effects of heavy metals and for predicting their toxic effects on the cells [63,64].

Our functional and molecular results have showed that the immunolocalization of MT protein and the expression of *mt* mRNA were induced after exposure to HgCl_2_ in *Danio rerio* liver in a dose dependent way. The results of this work showed that the induction of MTs after exposure to the contaminant was significant for both experimental concentrations compared to the control group. Furthermore, the functional investigation and the *in situ* hybridization analysis revealed that the MT protein and the *mt* mRNA were located in the cytoplasm of hepatocytes. The MTs are usually localized in the cytoplasm of the cells, but can be transferred into the nucleus according to the cell cycle changes [65]. Analysis of the localization of both *mt* mRNA and MT protein is important to clarify the physiological function of MTs [66]. According to Woo and Lazo [67] the cytoplasm localization of MTs has a suppressive effect on the cytotoxicity induced by heavy metals and decreases the level of intracellular reactive oxygen.

Ivanina and his collaborators [68] suggested that the metal toxicity in the cells is greatly reduced once the toxicant bound the MTs. This kind of connection between the MTs and the metals prevents the heavy metal interaction with other cellular components such as enzymes, proteins, DNA and lipids. Furthermore, high levels of MTs give to the cell sufficient protection from the toxic effects of heavy metals such as oxidation, denaturation and misfolding of protein.

In our study, although the increased expression of MTs in the liver, histological and ultrastructural damage was severe thus suggesting that the high toxicity of mercury does not allow MTs to perform successfully the metal detoxification in the cells.

In this work functional changes have been correlated to morphological and ultrastructural perturbations induced in liver of *Danio rerio* after exposure to mercury chloride.

Our results provide valuable information for a more comprehensive understanding of the effects of inorganic mercury on fish liver supporting the strong relationship between morphological and functional biomarkers. Further studies are needed to better understand the dangerous effects of heavy metals on hepatic tissue in order to have a more comprehensive overview of the impact on aquatic biota.

## 4. Materials and Methods

### 4.1. Test Organism and Experimental Design

*Danio rerio* (84 healthy adults of both sexes of length 0.35 ± 0.5 cm and weight 0.43 ± 0.06 g) were purchased from a local shop of ornamental fishes. Prior to experiments, fishes were acclimatized to laboratory conditions for 2 weeks in two 80 L aquaria filled with continuously aerated dechlorinated water. The temperature was kept at 25 ± 0.5 °C under a 12/12-h light-dark photoperiod. The fishes were fed every 2 days during the acclimation period with commercial fish food.

Zebrafish were exposed in a static system for 96 h to two mercury chloride (HgCl_2_; Sigma-Aldrich Chemical Co., St. Louis, MO, USA) concentrations of 7.7 and 38.5 μg/L; these concentrations correspond to 10% and 50% of the median lethal concentration at 96 h (LC50_96_) [69]. For each experimental unit, 14 animals of comparable body dimension were randomly chosen and assigned to 30 L constantly aerated glass tanks. Animals from the control group were kept in aged tap water. During experimental period animals were not fed and temperature, photoperiod, and aeration were kept under the same conditions used during the acclimation. Each treatment was conducted in duplicate.

The liver was removed after 96 h of exposure. Fishes from both control and exposed groups were anesthetized with tricaine methane sulfonate MS 222 (Sigma-Aldrich Chemical Co., St. Louis, MO, USA). Animal care, killing and experiments were supervised according to the European Convention for the Protection of Vertebrate Animals used for Experimental and other Scientific Purposes (Council of Europe No. 123, Strasbourg, 1985).

### 4.2. Light Microscopy and Transmission Electron Microscopy

The excised livers, cut into small pieces, were immediately fixed by direct immersion in 4% glutaraldehyde (Electron Microscopy Sciences, Hatfield, PA, USA) in phosphate-buffered saline (PBS 0.1 M, pH 7.2) for 48 h at 4 °C. After post-fixation for 2 h with osmium tetroxide (1% in the same buffer) and dehydration in graded ethanol, specimens for light microscopy (LM) and transmission electron microscopy (TEM) were embedded in epoxy resin (Araldite 502/Embed 812, Electron Microscopy Sciences). Semi-thin sections (1 μm) were stained with toluidine blue; these sections were observed and photographed by a LM LEITZ Dialux EB 20 (Leica Microsystems, Wetzlar, Germany). Ultrathin sections (800 Å) were stained with uranyl acetate replacement and contrasted using lead citrate (Electron Microscopy Sciences), then observed by a JEOL 1011 electron microscope (JEOL, Inc., Peabody, MA, USA).

### 4.3. Immunohistochemistry

For the immunohistochemical study, samples were fixed in Bouin solution for 48 h and then dehydrated in an increasing series of ethanol. The samples were xylene-cleared and embedded in paraffin wax (mean fusion point of 56 °C). Tissue sections (8 μm) were cut and mounted on slides. On deparaffinized and hydrated sections it was applied the indirect immunofluorescence technique [70]. To block non-specific sites, the slides were washed three times in PBS (pH 7.4) and then the sections were incubated for 10 min in a moist chamber with 20% normal goat serum. Unwashed slides were incubated overnight at 4 °C with a mouse monoclonal anti-metallothionein antibody (Stressgen Biotechnologies Corporation, Victoria, BC, Canada) at working dilutions of 1:100. The slides were washed several times with PBS and incubated with fluorescein isothiocyanate-conjugated γ-globulin goat anti-mouse (1:50, Sigma-Aldrich) for 30 min at room temperature in the dark. To visualize nuclei, the slides were stained with propidium iodide (a marker of nucleic acids) (1:200, Sigma-Aldrich), washed briefly with PBS and then mounted. Observations were performed by using a Leica TCS SP2 Confocal Laser Scanning Microscope (Leica Microsystems).

### 4.4. Mt mRNA in situ Hybridization

*In situ* hybridization of *mt* mRNA was performed according to Ebbesson and colleagues [71]. Samples of liver tissue were fixed in 4% paraformaldehyde in 0.1 M Sørensens phosphate buffer (28 mM NaH_2_PO_4_, pH 7.2) for 48 h.

After several washes in PBS (3 × 20 min), the tissue was cryoprotect by incubation in 25% sucrose (in PBS at 4 °C overnight), embedded in Tissue-Tek O.C.T. Compound (Sakura Fintek, Zoeterwoude, the Netherlands) and stored at −80 °C. Cryosectioning was performed using a Leica CM 1850 cryostat (Leica Microsystems). Collected transversal sections of livers (12 μm) were dried at 60 °C for 10 min before processing. Using a digoxigenin (DIG)-RNA labelling mix (Roche Diagnostics, Mannheim, Germany) the digoxigenin labelled RNA probes was arranged.

The *in situ* hybridization probe was 540 nucleotides long. The gene was cloned using the following primers: forward 5′-CCC AAG CTT ATT TCT AAG GAA CTT TCA AGC-3′ and reverse 5′-CCG CTC GAG TAA ATA CCA CCA TTT ATT TTA G-3′ according to Chen and colleagues [72]. The quality and quantity of the synthesized riboprobes were assessed by agarose gel electrophoresis.

The cryostat sections were kept at room temperature for 1 h and then placed at 65 °C for 10 min. Before the rehydration in ethanol series (from 95% to 50%) the sections were washed with 2× SSC, incubated in 10 μg/mL of proteinase K solution in 0.1 M Tris–HCl (pH 8.0) for 3.5 min. After a post-fixation in 4% paraformaldehyde in PBS for 5 min sections were washed in PBS (2 times for 2 min), and then incubated for 3 min in triethanolamine (TEA, 0.1 M pH 8.0; Sigma-Aldrich) and acetylated for 10 min with 0.25% acetic anhydride (Sigma-Aldrich) in 0.1 M TEA.

Before dehydration in ethanol the slides were washed in 2× SSC (1 min). Section were then air dried for 1 h. To each section were added 200 ng of digoxigenin labelled probe in 100 μL of hybridization solution [10 mM Tris-HCl, 300 mM NaCl, 20 mM of ethylenediaminetetraacetic acid—EDTA, 0.2% tween-20, 1% blocking solution (Roche Diagnostics), 0.1% dextran sulfate (Sigma-Aldrich), 50% deionized formamide (Sigma-Aldrich)]. The sections were covered with hybri-slips (Sigma-Aldrich) and incubated in a humid chamber for 16 h at 65 °C. As control for non-specific staining a sense probe was used.

After hybridization, the sections were sequentially incubated in: 2× SSC for 30 min (2 times), 50% deionized formamide in 2× SSC at 65 °C, 2× SSC at 37 °C for 10 min (2 times).

Then section were washed with RNase A (0.02 mg/mL, Sigma-Aldrich) (20 min at 37 °C and then 20 min at 65 °C). The slides were blocked with 2% blocking solution in 2× SSC with 0.05% Triton X-100 for 1 h and then incubated overnight in a humid chamber with alkaline phosphatase conjugated sheep anti-DIG goat antibody (1:2000, Roche Diagnostics).

The slides were washed in maleate buffer (2 times for 10 min) and finally the signal was visualized by incubation with freshly prepared visualization buffer + 100 mM Tris-HCl, 100 mM NaCl, (pH 9.5). The color reaction with chromogen substrate (3.4 μL of nitroblue tetrazolium, 3.5 μL of 5-bromo-4-chloro-3-indoylphosphate (Roche Diagnostics) in visualization buffer) was carried out for 3 h in darkness at room temperature. The reaction was terminated with stop solution (10 mM Tris-HCl, 1 mM EDTA, 150 mM NaCl, pH 8.0) and tissue was mounted with 70% glycerol. Photographs were taken using a LM LEITZ Dialux EB 20.

### 4.5. Quantitative RT-PCR

Extraction of total RNAs from 30 mg of liver tissue, was performed using the PureLink RNA Mini Kit (Thermo Fisher Scientific, Waltham, MA, USA) according to the manufacturer’s instructions. To check the extraction quality, all produced RNAs were electrophoresed on a 1% agarose-formaldehyde gel. RNAs concentration was determined by spectrophotometry. For each exposure condition five replicates were performed. First-strand cDNA was synthesized from 2 μg of total RNA using the High capacity RNA to cDNA Kit (Applied Biosystems, Foster City, CA, USA) according to the manufacturer’s instructions. The cDNA was conserved at −20 °C until it use in a real-time PCR reaction. The gene used in our study is the metal-regulatory transcription factor 1 (mtf1, accession number NM_152981.1) and two reference genes, glyceraldehyde-3-phosphate dehydrogenase (gapdh, accession number NM_001115114.1) and actin beta 1(actb1; accession number NM_131031.1). The amplification of cDNA was checked using the TaqMan Gene Expression Assays. Real-time PCR reactions were performed in a Light Cycler (Applied Biosystems StepOn, Real-Time PCR System, Foster City, CA, USA) following the manufacturer’s instructions (one cycle at 50 °C for 2 min, 95 °C for 10 min and 40 amplification cycles at 95 °C for 15 s, 60 °C for 1 min). Each 20 μL reaction contained 2 μL of reverse transcribed product template, 10 μL of master mix (TaqMan Universal Master Mix II) (Applied Biosystems), 1 μL of assay mix (TaqMan Gene Expression Assay) including a pair of unlabeled gene specific PCR primers and a TaqMan probe with a FAM dye label on the 5′, a minor groove binder (MGB) and non-fluorescent quencher (NFQ) on the 3′ end) and 7 μL of H_2_O RNase-free.

The reaction specificity was determined for each reaction from the dissociation curve of the PCR product. Relative quantification of each gene expression level was normalized according to the average of the two housekeeping gene expression levels (*actb1* and *gapdh*). Relative mRNA expression of the gene was generated using the 2^−Δ*C*t^ method [73].

### 4.6. Statistical Analysis

The effect of HgCl_2_ exposure on MT gene expression in the liver tissue of *Danio rerio* was performed using Graph Pad Prism 5.00 (GraphPad Software Inc., San Diego, CA, USA) at significance level of 0.05. Assumptions of normality and homoscedasticity were tested with KS normality test and Bartlett’s test, respectively. The effect of HgCl_2_ exposure was compared using one-way ANOVA followed by Bonferroni’s Multiple Comparison Test.

## References

[1] Liu G., Cai Y., O’Driscoll N., Feng X., Jiang G., Liu G., Cai Y., O’Driscoll N. (2012). Overview of mercury in the environment. Environmental Chemistry and Toxicology of Mercury.

[2] Chen C.W., Chen C.F., Dong C.D. (2012). Distribution and accumulation of mercury in sediments of Kaohsiung River Mouth, Taiwan. APCBEE Procedia.

[3] Driscoll C.T., Mason R.P., Chan H.M., Jacob D.J., Pirrone N. (2013). Mercury as a global pollutant: Sources, pathways, and effects. Environ. Sci. Technol..

[4] Wiener J.G. (2013). Mercury exposed: Advances in environmental analysis and ecotoxicology of a highly toxic metal. Environ. Toxicol. Chem..

[5] WHO (World Health Organization) Mercury and health, 2013. http://www.who.int/mediacentre/factsheets/fs361/en/.

[6] Sharma M.K., Sharma A., Kumar A., Kumar M. (2007). Evaluation of protective efficacy of *Spirulina fusiformis* against mercury induced nephrotoxicity in Swiss albino mice. Food Chem. Toxicol..

[7] Clarkson T.W., Magos L., Myers G.J. (2003). The toxicology of mercury—Current exposures and clinical manifestations. N. Engl. J. Med..

[8] Bando I., Reus M.I.S., Andrés D., Cascales M. (2003). Endogenous antioxidant defence system in rat liver following mercury chloride oral intoxication. J. Biochem. Mol. Toxic..

[9] Gentès S., Maury-Brachet R., Feng C., Pedrero Z., Tessier E., Legeay A., Mesmer-Dudons N., Baudrimont M., Maurice L., Amouroux D. (2015). Specific effects of dietary methylmercury and inorganic mercury in zebrafish (*Danio rerio*) determined by genetic, histological, and metallothionein responses. Environ. Sci. Technol..

[10] Rao M.V., Chhunchha B. (2010). Protective role of melatonin against the mercury induced oxidative stress in the rat thyroid. Food Chem. Toxicol..

[11] Jaishankar M., Tseten T., Anbalagan N., Mathew B.B., Beeregowda K.N. (2014). Toxicity, mechanism and health effects of some heavy metals. Interdiscip. Toxicol..

[12] Senger M.R., Rosemberg D.B., Seibt K.J., Dias R.D., Bogo M.R., Bonan C.D. (2010). Influence of mercury chloride on adenosine deaminase activity and gene expression in zebrafish (*Danio rerio*) brain. Neurotoxicology.

[13] Hassan S.A., Moussa E.A., Abbott L.C. (2012). The effect of methylmercury exposure on early central nervous system development in the zebrafish (*Danio rerio*) embryo. J. Appl. Toxicol..

[14] Ho N.Y., Yang L., Legradi J., Armant O., Takamiya M., Rastegar S., Strähle U. (2013). Gene responses in the central nervous system of zebrafish embryos exposed to the neurotoxicant methyl mercury. Environ. Sci. Technol..

[15] Cambier S., Benard G., Mesmer-Dudons N., Gonzalez P., Rossignol R., Brethes D., Bourdineaud J.P. (2009). At environmental doses, dietary methylmercury inhibits mitochondrial energy metabolism in skeletal muscles of the zebrafish (*Danio rerio*). Int. J. Biochem. Cell B.

[16] Cambier S., Gonzalez P., Durrieu G., Maury-Brachet R., Boudou A., Bourdineaud J.P. (2009). Serial analysis of gene expression in the skeletal muscles of zebrafish fed with a methylmercury-contaminated diet. Environ. Sci. Technol..

[17] Aboud O.A.S.A. (2010). Impact of pollution with lead, mercury and cadmium on the immune response of *Oreochromis niloticus*. N.Y. Sci. J..

[18] Penglase S., Hamre K., Ellingsen S. (2014). Selenium and mercury have a synergistic negative effect on fish reproduction. Aquat. Toxicol..

[19] Berg K., Puntervoll P., Valdersnes S., Goksøyr A. (2010). Responses in the brain proteome of Atlantic cod (*Gadus morhua*) exposed to methylmercury. Aquat. Toxicol..

[20] Berntssen M.H., Aatland A., Handy R.D. (2003). Chronic dietary mercury exposure causes oxidative stress, brain lesions, and altered behaviour in Atlantic salmon (*Salmo salar*) parr. Aquat. Toxicol..

[21] Brandão F., Cappello T., Raimundo J., Santos M.A., Maisano M., Mauceri A., Pacheco M., Pereira P. (2015). Unravelling the mechanisms of mercury hepatotoxicity in wild fish (*Liza aurata*) through a triad approach: Bioaccumulation, metabolomic profiles and oxidative stress. Metallomics.

[22] Ung C.Y., Lam S.H., Hlaing M.M., Winata C.L., Korzh S., Mathavan S., Gong Z. (2010). Mercury-induced hepatotoxicity in zebrafish: *In vivo* mechanistic insights from transcriptome analysis, phenotype anchoring and targeted gene expression validation. BMC Genom..

[23] Gonzalez P., Dominique Y., Massabuau J.C., Boudou A., Bourdineaud J.P. (2005). Comparative effects of dietary methylmercury on gene expression in liver, skeletal muscle, and brain of the zebrafish (*Danio rerio*). Environ. Sci. Technol..

[24] Mela M., Neto F.F., Yamamoto F.Y., Almeida R., Grötzner S.R., Ventura D.F., de Oliveira Ribeiro C.A. (2014). Mercury distribution in target organs and biochemical responses after subchronic and trophic exposure to Neotropical fish *Hoplias malabaricus*. Fish Physiol. Biochem..

[25] Bleau H., Daniel C., Chevalier G., van Tra H., Hontela A. (1996). Effects of acute exposure to mercuric chloride and methylmercury on plasma cortisol, T3, T4, glucose, and liver glycogen in rainbow trout (*Oncorhynchus mykiss*). Aquat. Toxicol..

[26] Jesus T.B.D., Almeida P.G.A.D., Vergílio C.D.S., Machado A.L.D.S., Carvalho C.E.V.D. (2011). Acute intraperitoneal mercury chloride contamination and distribution in liver, muscle and gill of a neotropical fish *Hoplias malabaricus* (BLOCK, 1794). Braz. Arch. Biol. Technol..

[27] Roy George K., Malini N.A., Sandhya Rani G.O. (2012). Biochemical changes in liver and muscle of the cichlid, *Oreochromis mossambicus* (Peters, 1852) exposed to sub-lethal concentration of mercuric chloride. Indian J. Fish..

[28] Karlsen O.A., Sheehan D., Goksøyr A. (2014). Alterations in the Atlantic cod (*Gadus morhua*) hepatic thiol-proteome after methylmercury exposure. J. Toxicol. Environ. Heal. A.

[29] Yadetie F., Karlsen O.A., Lanzén A., Berg K., Olsvik P., Hogstrand C., Goksøyr A. (2013). Global transcriptome analysis of Atlantic cod (*Gadus morhua*) liver after *in vivo* methylmercury exposure suggests effects on energy metabolism pathways. Aquat. Toxicol..

[30] Mela M., Randi M.A.F., Ventura D.F., Carvalho C.E.V., Pelletier E., de Oliveira Ribeiro C.A. (2007). Effects of dietary methylmercury on liver and kidney histology in the neotropical fish *Hoplias malabaricus*. Ecotoxicol. Environ. Saf..

[31] De Oliveira Ribeiro C.A., Belger L., Pelletier E., Rouleau C. (2002). Histopathological evidence of inorganic mercury and methyl mercury toxicity in the arctic charr (*Salvelinus alpinus*). Environ. Res..

[32] Régine M.B., Gilles D., Yannick D., Alain B. (2006). Mercury distribution in fish organs and food regimes: Significant relationships from twelve species collected in French Guiana (Amazonian basin). Sci. Total Environ..

[33] De Oliveira Ribeiro C.O., Pelletier E., Pfeiffer W.C., Rouleau C. (2000). Comparative uptake, bioaccumulation, and gill damages of inorganic mercury in tropical and nordic freshwater fish. Environ. Res..

[34] Elia A.C., Galarini R., Taticchi M.I., Dörra A.J.M., Mantilacci L. (2003). Antioxidant responses and bioaccumulation in *Ictalurus melas* under mercury exposure. Ecotoxicol. Environ. Saf..

[35] Hill A.J., Teraoka H., Heideman W., Peterson R.E. (2005). Zebrafish as a model vertebrate for investigating chemical toxicity. Toxicol. Sci..

[36] Korbas M., Blechinger S.R., Krone P.H., Pickering I.J., George G.N. (2008). Localizing organomercury uptake and accumulation in zebrafish larvae at the tissue and cellular level. Proc. Natl. Acad. Sci. USA.

[37] Korbas M., Krone P.H., Pickering I.J., George G.N. (2010). Dynamic accumulation and redistribution of methylmercury in the lens of developing zebrafish embryos and larvae. J. Biol. Inorg. Chem..

[38] Korbas M., MacDonald T.C., Pickering I.J., George G.N., Krone P.H. (2011). Chemical form matters: Differential accumulation of mercury following inorganic and organic mercury exposures in zebrafish larvae. ACS Chem. Biol..

[39] Brunelli E., Talarico E., Corapi B., Perrotta I., Tripepi S. (2008). Effects of a sublethal concentration of sodium lauryl sulphate on the morphology and Na^+^/K^+^ ATPase activity in the gill of the ornate wrasse (*Thalassoma pavo*). Ecotoxicol. Environ. Saf..

[40] Brunelli E., Mauceri A., Maisano M., Bernabò I., Giannetto A., de Domenico E., Corapi B., Tripepi S., Fasulo S. (2011). Ultrastructural and immunohistochemical investigation on the gills of the teleost, *Thalassoma pavo* L., exposed to cadmium. Acta Histochem..

[41] Ivanković D., Pavičić J., Erk M., Filipović-Marijić V., Raspor B. (2005). Evaluation of the *Mytilus galloprovincialis* Lam. Digestive gland metallothionein as a biomarker in a long-term field study: Seasonal and spatial variability. Mar. Pollut. Bull..

[42] Sinaie M., Bastami K.D., Ghorbanpour M., Najafzadeh H., Shekari M., Haghparast S. (2010). Metallothionein biosynthesis as a detoxification mechanism in mercury exposure in fish, spotted scat (*Scatophagus argus*). Fish Physiol. Biochem..

[43] Hamza-Chaffai A., Amiard J.C., Pellerin J., Joux L., Berthet B. (2000). The potential use of metallothionein in the clam *Ruditapes decussatus* as a biomarker of *in situ* metal exposure. Comp. Biochem. Phys. C.

[44] De Boeck G., Ngo T.T.H., van Campenhout K., Blust R. (2003). Differential metallothionein induction patterns in three freshwater fish during sublethal copper exposure. Aquat. Toxicol..

[45] Bebianno M.J., Santos C., Canário J., Gouveia N., Sena-Carvalho D., Vale C. (2007). Hg and metallothionein-like proteins in the black scabbardfish *Aphanopus carbo*. Food Chem. Toxicol..

[46] Braunbeck T., Storch V., Bresch H. (1990). Species-specific reaction of liver ultrastructure in zebrafish (*Brachydanio rerio*) and trout (*Salmo gairdneri*) after prolonged exposure to 4-chloroaniline. Arch. Environ. Contam. Toxicol..

[47] Yao Y., Lin J., Yang P., Chen Q., Chu X., Gao C., Hu J. (2012). Fine structure, enzyme histochemistry, and immunohistochemistry of liver in zebrafish. Anat. Rec..

[48] Paris-Palacios S., Biagianti-Risbourg S., Vernet G. (2000). Biochemical and (ultra) structural hepatic perturbations of *Brachydanio rerio* (Teleostei, Cyprinidae) exposed to two sublethal concentrations of copper sulfate. Aquat. Toxicol..

[49] Gogbold D.L., Watras C.J., Huckabee J.W. (1994). Mercury in forest ecosystem: Risk and research needs. Mercury Pollution: Integration and synthesis.

[50] Bais U.E., Lokhande M.V. (2012). Effect of cadmium chloride on histopathological changes in the freshwater fish *Ophiocephalus striatus* (Channa). Int. J. Zool. Res..

[51] Khidr B.M., Mekkawy I.A., Harabawy A.S., Ohaida A.S. (2012). Effect of lead nitrate on the liver of the cichlid fish (*Oreochromis niloticus*): A light microscope study. Pak. J. Biol. Sci..

[52] Drevnick P.E., Roberts A.P., Otter R.R., Hammerschmidt C.R., Klaper R., Oris J.T. (2008). Mercury toxicity in livers of northern pike (*Esox lucius*) from Isle Royale, USA. Comp. Biochem. Phys. C.

[53] De Lisle R.C., Sarras M.P., Hidalgo J., Andrews G.K. (1996). Metallothionein is a component of exocrine pancreas secretion: Implications for zinc homeostasis. Am. J. Physiol Cell Physiol..

[54] Pearce L.L., Wasserloos K., Croix C.M.S., Gandley R., Levitan E.S., Pitt B.R. (2000). Metallothionein, nitric oxide and zinc homeostasis in vascular endothelial cells. J. Nutr..

[55] Ecker D.J., Butt T.R., Sternberg E.J., Neeper M.P., Debouck C., Gorman J.A., Crooke S.T. (1986). Yeast metallothionein function in metal ion detoxification. J. Biol. Chem..

[56] Huang P.C., Morris S., Dinman J., Pine R., Smith B. (1987). Role of metallothionein in detoxification and tolerance to transition metals. Exp. Suppl..

[57] Li X., Chen H., Epstein P.N. (2004). Metallothionein protects islets from hypoxia and extends islet graft survival by scavenging most kinds of reactive oxygen species. J. Biol. Chem..

[58] Anderson R.S., Patel K.M., Roesijadi G. (1999). Oyster metallothionein as an oxyradical scavenger: Implications for hemocyte defense responses. Dev. Comp. Immunol..

[59] Morris C.A., Stürzenbaum S., Nicolaus B., Morgan A.J., Harwood J.L., Kille P., Klaassen C.D. (1999). Identification and characterisation of metallothioneins from environmental indicator species as potential biomonitors. Metallothionein IV.

[60] Hamza-Chaffai A., Amiard J.C., Cosson R.P. (1999). Relationship between metallothioneins and metals in a natural population of the clam *Ruditapes decussatus* from Sfax coast: A non-linear model using Box-Cox transformation. Comp. Biochem. Phys. C.

[61] Cosson R.P., Amiard J.C., Lagadic L., Caquet T., Amiard J.C., Ramade F. (2000). Use of metallothionein as biomarkers of exposure to metals. Use of Biomarkers for Environmental Quality Assessment.

[62] Geffard A., Amiard-Triquet C., Amiard J.C. (2005). Do seasonal changes affect metallothionein induction by metals in mussels, *Mytilus edulis*?. Ecotoxicol. Environ. Saf..

[63] Knapen D., Reynders H., Bervoets L., Verheyen E., Blust R. (2007). Metallothionein gene and protein expression as a biomarker for metal pollution in natural gudgeon populations. Aquat. Toxicol..

[64] Chen L., Ma L., Bai Q., Zhu X., Zhang J., Wei Q., Li D., Gao C., Li J., Zhang Z. (2014). Heavy metal-induced metallothionein expression is regulated by specific protein phosphatase 2A complexes. J. Biol. Chem..

[65] Thophon S., Pokethitiyook P., Chalermwat K., Upatham E.S., Sahaphong S. (1993). Ultrastructural alterations in the liver and kidney of white sea bass, *Lates calcarifer*, in acute and subchronic cadmium exposure. Environ. Toxicol..

[66] Sato M., Kondoh M. (2002). Recent studies on metallothionein: Protection against toxicity of heavy metals and oxygen free radicals. Tohoku J. Exp. Med..

[67] Woo E.S., Lazo J.S. (1997). Nucleocytoplasmic functionality of metallothionein. Cancer Res..

[68] Ivanina A.V., Cherkasov A.S., Sokolova I.M. (2008). Effects of cadmium on cellular protein and glutathione synthesis and expression of stress proteins in eastern oysters, *Crassostrea virginica* Gmelin. J. Exp. Biol..

[69] Vutukuru S.S., Basani K. (2013). Acute effects of mercuric chloride on glycogen and protein content of Zebra fish, *Danio rerio*. J. Environ. Biol..

[70] Coons A.H., Leduc E.H., Connolly J.M. (1955). Studies on antibody. I. A method for thehistochemical demonstration of specific antibody and its application to a studyof the hyperimmune rabbit. J. Exp. Med..

[71] Ebbesson L.O.E., Nilsen T.O., Helvik J.V., Tronci V., Stefansson S.O. (2011). Corticotropin-releasing factor neurogenesis during midlife development in salmon: Genetic, environmental and thyroid hormone regulation. J. Neuroendocrinol..

[72] Chen W.Y., John J.A.C., Lin C.H., Lin H.F., Wu S.C., Lin C.H., Chang C.Y. (2004). Expression of metallothionein gene during embryonic and early larval development in zebrafish. Aquat. Toxicol..

[73] Livak K.J., Schmittgen T.D. (2001). Analysis of relative gene expression data using real-time quantitative PCR and the 2^−ΔΔ*C*t^ method. Methods.

